# Supplementary material for: Photosynthesis, Phytohormone Signaling and Sugar Catabolism in the Culm Sheaths of Phyllostachys edulis
Source: Plants (Basel). 2022 Oct 27;11(21):2866. doi: 10.3390/plants11212866 (PMC9655093; doi:10.3390/plants11212866)

Figure S1. Diurnal variation of gas exchange parameters in bamboo shoot sheath blades. (A) Pn: net photosynthetic rate. (B) CO_{2int}: intercellular CO_2 concentration. (C) E: transpiration rate. (D) gs: stomatal conductance. (E) WUE: water-use efficiency. The experiments were repeated three times. $P < 0.05$.

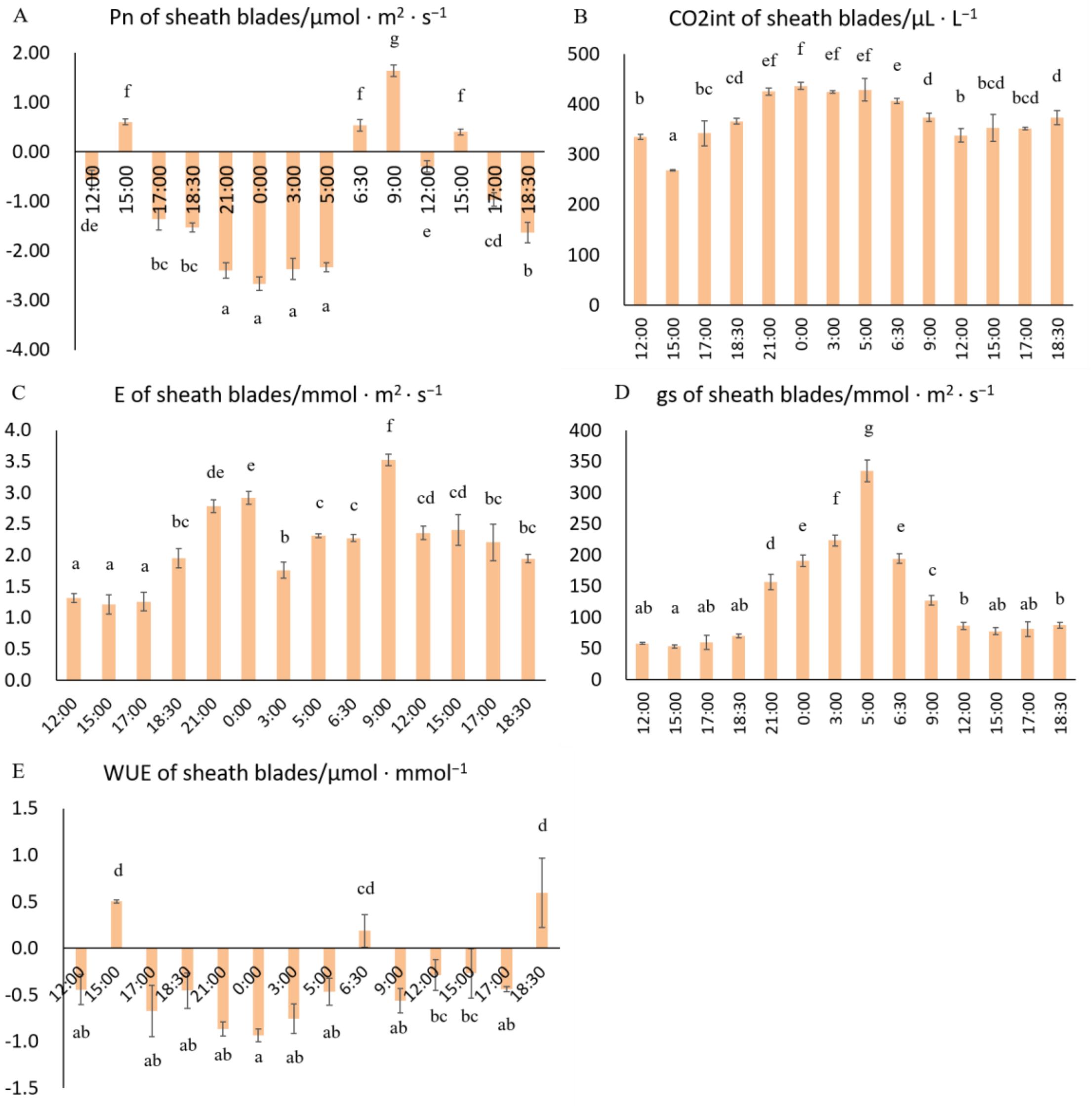

Supplement: Supplementary file 1 [file plants-11-02866-s001.zip › Figure S1.pdf]
