# Supplementary material for: Photosynthesis, Phytohormone Signaling and Sugar Catabolism in the Culm Sheaths of Phyllostachys edulis
Source: Plants (Basel). 2022 Oct 27;11(21):2866. doi: 10.3390/plants11212866 (PMC9655093; doi:10.3390/plants11212866)
Supplement: Supplementary file 1 [file plants-11-02866-s001.zip › Figure S2.pdf]

Figure S2. Anatomy and statistics of sheath blades. (A) Sheath blades at the base of the bamboo shoot. (B) Sheath blades in the middle of the bamboo shoot. (C) Sheath blades at the upper part of the bamboo shoot. fc, fusoid cells; ph, phloem; pc, parenchymal cells; sc, sclerenchymal cells; xy, xylem duct; ec, epidermal cells. Bar = 100 μm. (D) Vascular bundle sectional area of sheath blades. (E) Phloem sectional area of sheath blades. (F) Xylem duct sectional area of sheath blades. (G) Epidermal cell thickness of sheath blades. (H) Distance between vascular bundles of sheath blades. $P < 0.05$.

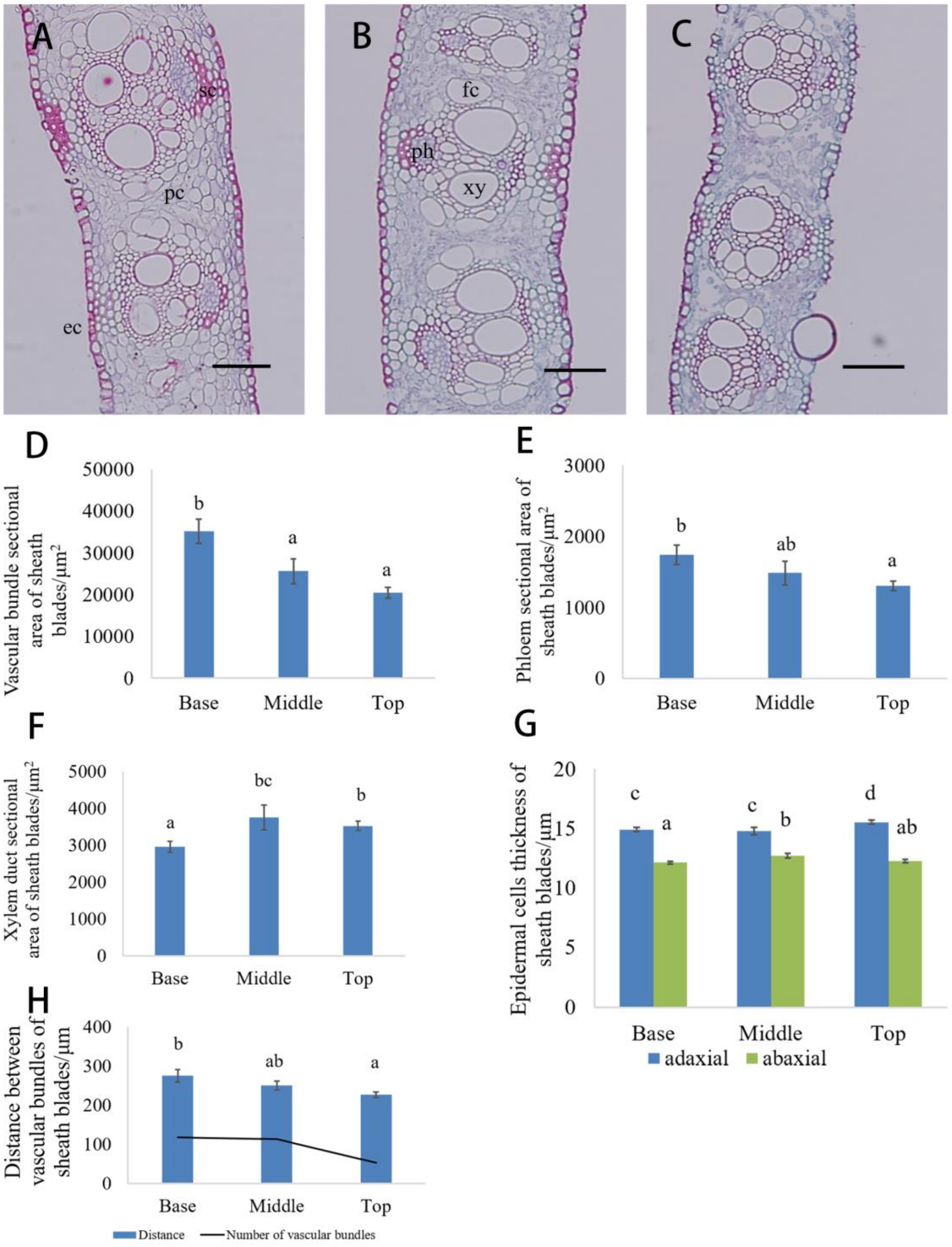
A
sc
pc
ec
B
fc
ph
xy
C
D
Vascular bundle sectional area of sheath blades/μm²
50000
40000
30000
20000
10000
0
b
a
a
Base
Middle
Top
E
Phloem sectional area of sheath blades/μm²
3000
2000
1000
0
b
ab
a
Base
Middle
Top
F
Xylem duct sectional area of sheath blades/μm²
5000
4000
3000
2000
1000
0
a
bc
b
Base
Middle
Top
G
Epidermal cells thickness of sheath blades/μm
20
15
10
5
0
c
a
c
b
d
ab
Base
Middle
Top
adaxial
abaxial
H
Distance between vascular bundles of sheath blades/μm
400
300
200
100
0
b
ab
a
Base
Middle
Top
Distance
Number of vascular bundles
