# Supplementary material for: Photosynthesis, Phytohormone Signaling and Sugar Catabolism in the Culm Sheaths of Phyllostachys edulis
Source: Plants (Basel). 2022 Oct 27;11(21):2866. doi: 10.3390/plants11212866 (PMC9655093; doi:10.3390/plants11212866)

**Figure S3.** Acidic fuchsin staining tracer for vascular bundles. (A) Lateral observation. (B) Section observation. The red arrow indicates vascular bundles connecting the bamboo sheath to the bamboo shoot.

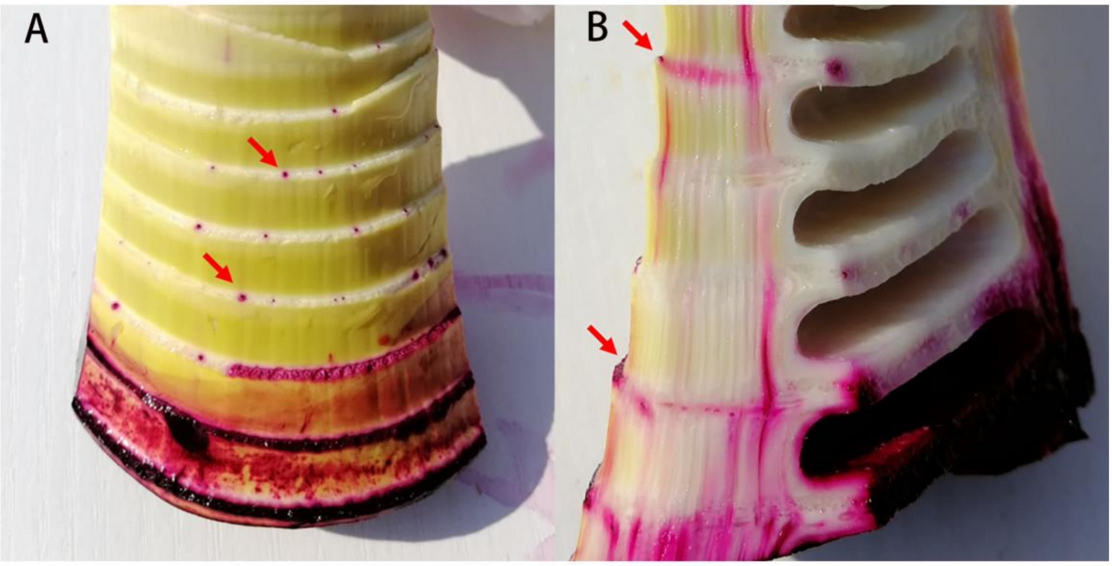

Supplement: Supplementary file 1 [file plants-11-02866-s001.zip › Figure S3.pdf]
