# Supplementary material for: Photosynthesis, Phytohormone Signaling and Sugar Catabolism in the Culm Sheaths of Phyllostachys edulis
Source: Plants (Basel). 2022 Oct 27;11(21):2866. doi: 10.3390/plants11212866 (PMC9655093; doi:10.3390/plants11212866)
Supplement: Supplementary file 1 [file plants-11-02866-s001.zip › Figure S4.pdf]

Figure S4. Sugar content in different sections of the culm sheath. (A) Glucose content. (B) Fructose content. (C) Sucrose content in bamboo sheaths. The 3–5 pieces of the bamboo sheath located at the base of bamboo shoot are labeled as “Base,” the 13–15 pieces of the bamboo sheath located in the middle of the bamboo shoot are labeled as “Middle,” and 23–25 pieces of the bamboo shoot sheath located at the upper part of the bamboo shoot are labeled as “Top.” For a single bamboo sheath, the sampling sites from bottom to top are “sheath base,” “sheath upper,” and “sheath blade.” The experiments were repeated three times. $P < 0.05$.

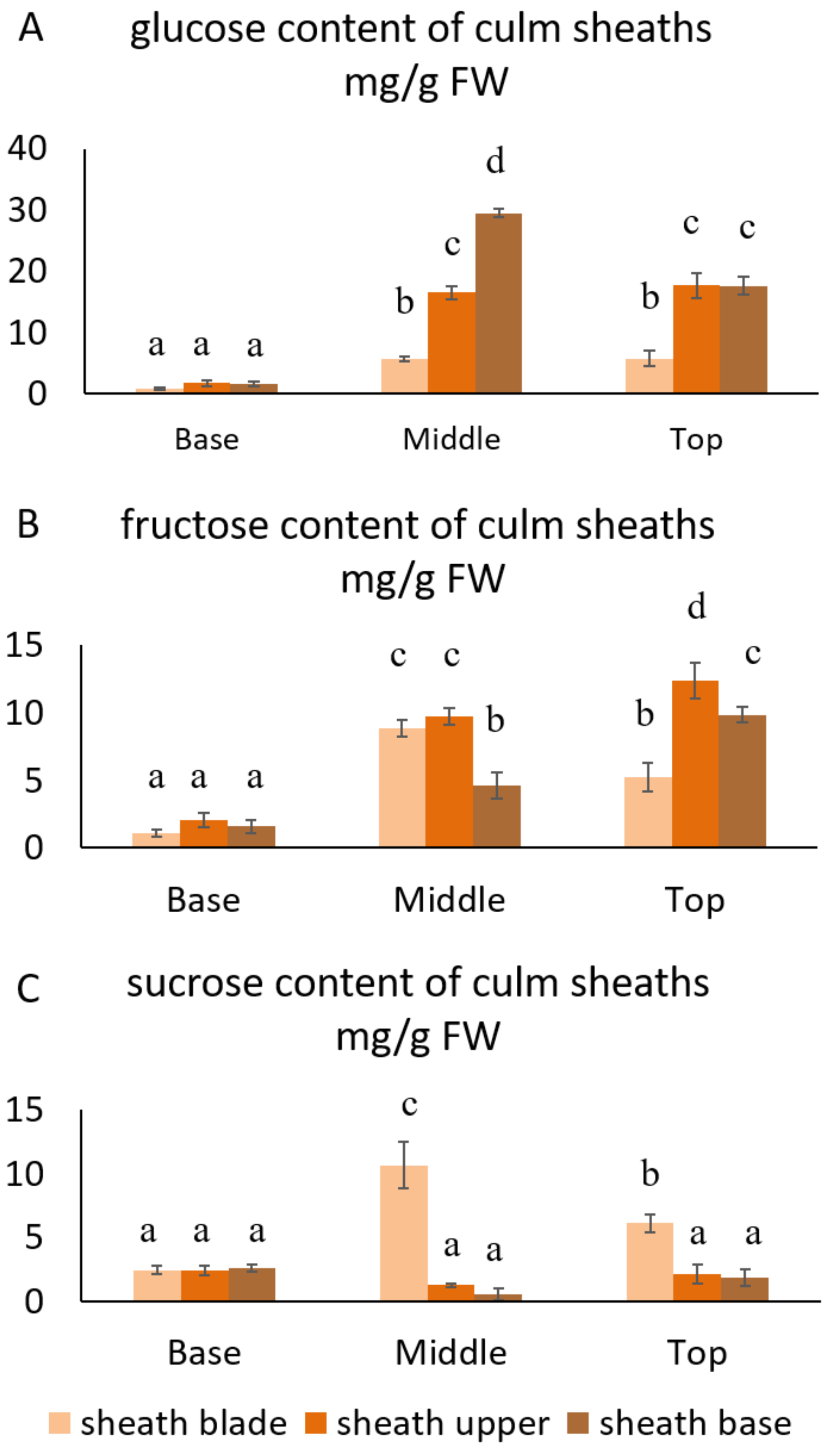
A
glucose content of culm sheaths
mg/g FW
40
30
20
10
0
a a a
b c d
b c c
Base
Middle
Top
B
fructose content of culm sheaths
mg/g FW
15
10
5
0
a a a
c c b
b d c
Base
Middle
Top
C
sucrose content of culm sheaths
mg/g FW
15
10
5
0
a a a
c a a
b a a
Base
Middle
Top
sheath blade
sheath upper
sheath base
