# Supplementary material for: Photosynthesis, Phytohormone Signaling and Sugar Catabolism in the Culm Sheaths of Phyllostachys edulis
Source: Plants (Basel). 2022 Oct 27;11(21):2866. doi: 10.3390/plants11212866 (PMC9655093; doi:10.3390/plants11212866)

**Figure S5.** KEGG enrichment and classification analysis of differentially expressed genes. A KEGG enrichment analysis of differentially expressed genes; B KEGG class analysis of differentially expressed genes.

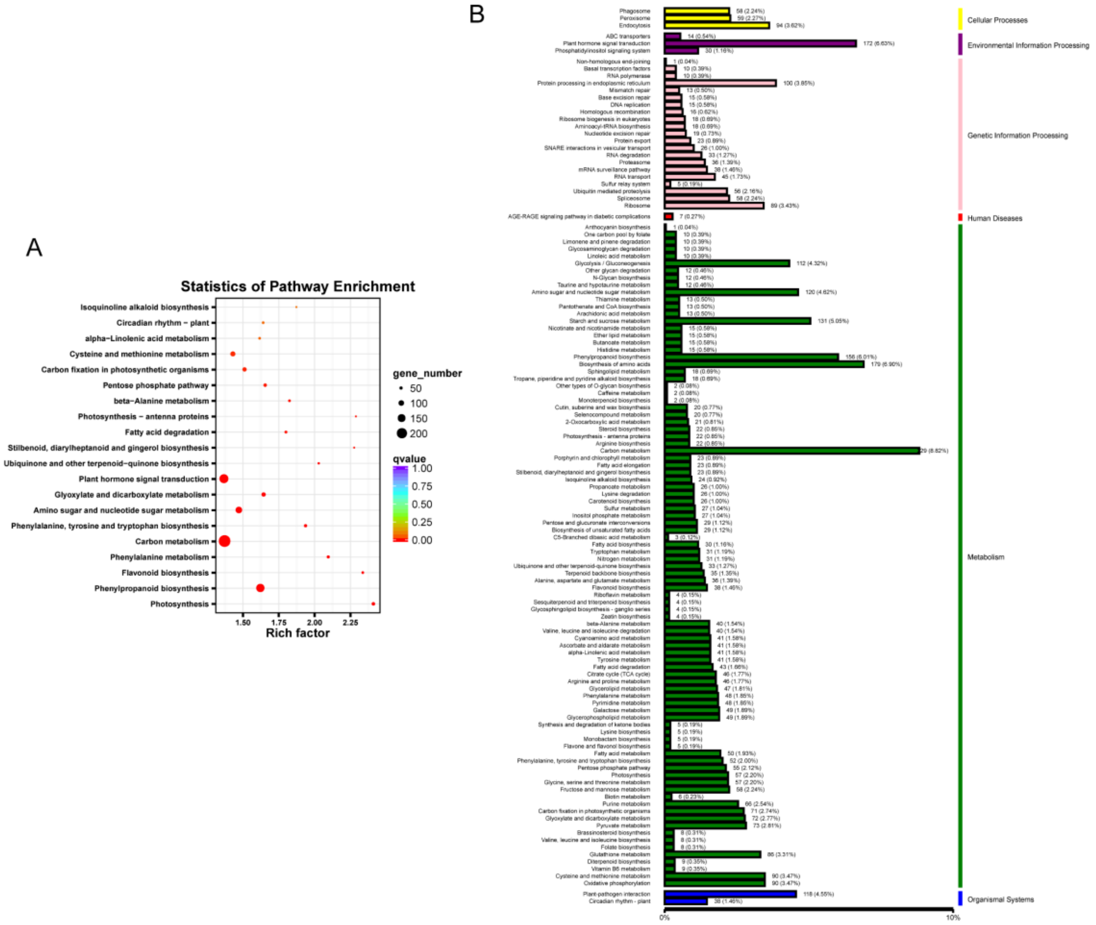

Supplement: Supplementary file 1 [file plants-11-02866-s001.zip › Figure S5.pdf]
