# Supplementary figures and images for: Photosynthesis, Phytohormone Signaling and Sugar Catabolism in the Culm Sheaths of Phyllostachys edulis
Source: Plants (Basel). 2022 Oct 27;11(21):2866. doi: 10.3390/plants11212866 (PMC9655093; doi:10.3390/plants11212866)

**Figure S6.** Heatmap of differentially expressed genes in photosynthetic signaling pathways.

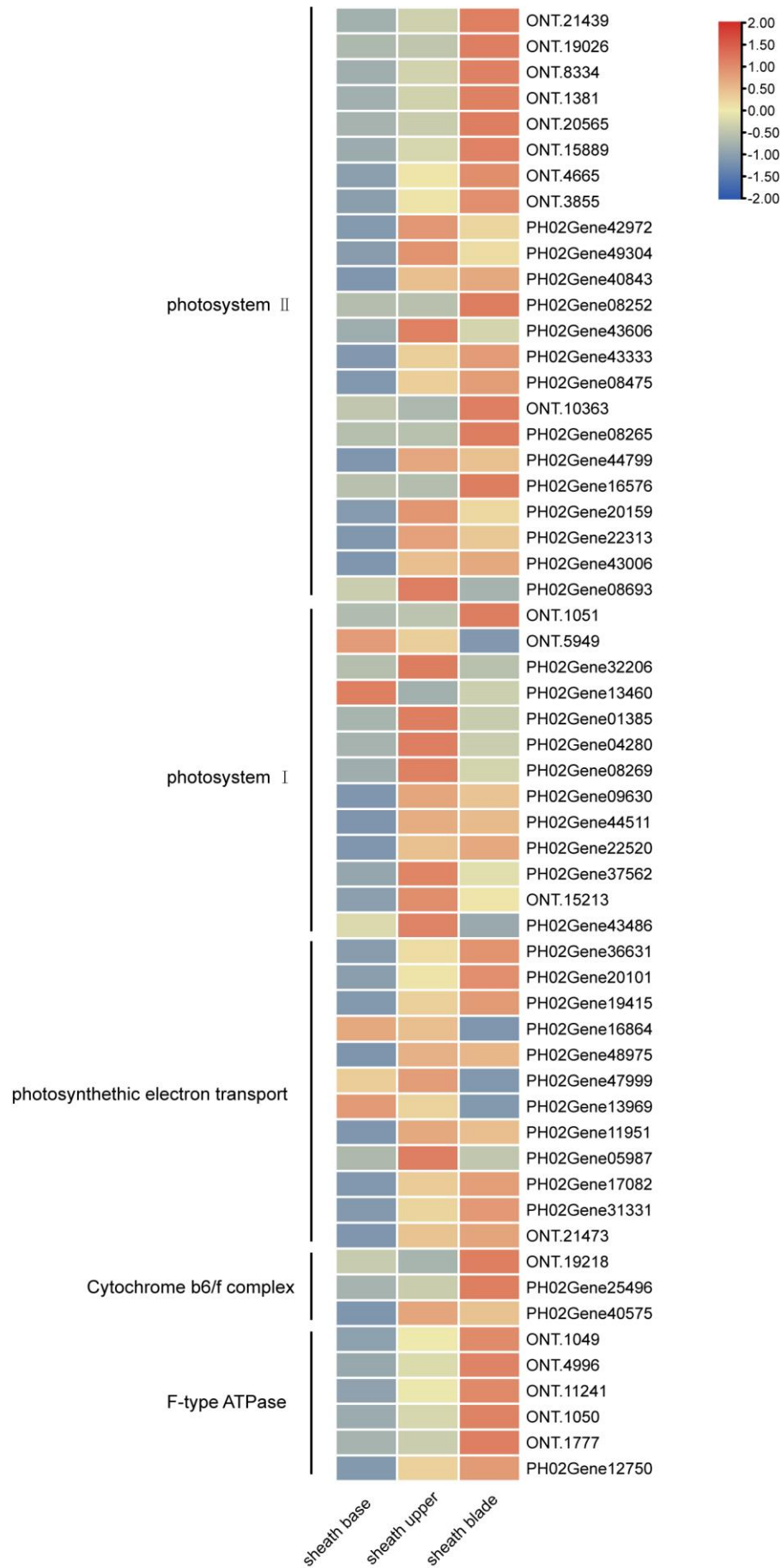

Supplement: Supplementary file 1 [file plants-11-02866-s001.zip › Figure S6.pdf]
